# Adaptive evolution of the spike gene of SARS coronavirus: changes in positively selected sites in different epidemic groups

**DOI:** 10.1186/1471-2180-6-88

**Published:** 2006-10-04

**Authors:** Chi-Yu Zhang, Ji-Fu Wei, Shao-Heng He

**Affiliations:** 1 Department of Biochemistry and Molecular Biology, Jiangsu University School of Medical Technology, Zhenjiang, Jiangsu 212001, China; 2 The First Affiliated Hospital of Nanjing Medical University, Nanjing, Jiangsu 210026, China

## Abstract

**Background:**

It is believed that animal-to-human transmission of severe acute respiratory syndrome (SARS) coronavirus (CoV) is the cause of the SARS outbreak worldwide. The spike (S) protein is one of the best characterized proteins of SARS-CoV, which plays a key role in SARS-CoV overcoming species barrier and accomplishing interspecies transmission from animals to humans, suggesting that it may be the major target of selective pressure. However, the process of adaptive evolution of S protein and the exact positively selected sites associated with this process remain unknown.

**Results:**

By investigating the adaptive evolution of S protein, we identified twelve amino acid sites (75, 239, 244, 311, 479, 609, 613, 743, 765, 778, 1148, and 1163) in the S protein under positive selective pressure. Based on phylogenetic tree and epidemiological investigation, SARS outbreak was divided into three epidemic groups: 02–04 interspecies, 03-early-mid, and 03-late epidemic groups in the present study. Positive selection was detected in the first two groups, which represent the course of SARS-CoV interspecies transmission and of viral adaptation to human host, respectively. In contrast, purifying selection was detected in 03-late group. These indicate that S protein experiences variable positive selective pressures before reaching stabilization. A total of 25 sites in 02–04 interspecies epidemic group and 16 sites in 03-early-mid epidemic group were identified under positive selection. The identified sites were different between these two groups except for site 239, which suggests that positively selected sites are changeable between groups. Moreover, it was showed that a larger proportion (24%) of positively selected sites was located in receptor-binding domain (RBD) than in heptad repeat (HR)1-HR2 region in 02–04 interspecies epidemic group (p = 0.0208), and a greater percentage (25%) of these sites occurred in HR1–HR2 region than in RBD in 03-early-mid epidemic group (p = 0.0721). These suggest that functionally different domains of S protein may not experience same positive selection in each epidemic group. In addition, three specific replacements (F360S, T487S and L665S) were only found between 03-human SARS-CoVs and strains from 02–04 interspecies epidemic group, which reveals that selective sweep may also force the evolution of S genes before the jump of SARS-CoVs into human hosts. Since certain residues at these positively selected sites are associated with receptor recognition and/or membrane fusion, they are likely to be the crucial residues for animal-to-human transmission of SARS-CoVs, and subsequent adaptation to human hosts.

**Conclusion:**

The variation of positive selective pressures and positively selected sites are likely to contribute to the adaptive evolution of S protein from animals to humans.

## Background

SARS is a new infectious disease that emerged in the Guangdong province of China in November 2002. It caused 8,096 infection cases including 774 deaths worldwide during its epidemic [1]. The causative pathogen of SARS was identified as a novel strain of human coronavirus, named as SARS-CoV, and its complete genome was sequenced in March 2003 [2-5]. In May 2003, SARS-CoVs were also isolated from a few Himalayan palm civets (*Paguma larvata*) and a raccoon dog (*Nyctereutes procyonoides*) in a food market in Shenzhen (Guangdong, China) [6]. These isolations provided the first evidence that wild animals could be reservoirs for SARS-CoV, and that the virus might be transmitted from animals to humans. The re-emergence of SARS in 2003–2004 in Guangdong, China confirmed that SARS-CoV was independently transmitted from animals to humans [7].

The S protein of SARS-CoV is composed of 1,255 amino acids, and is responsible for viral attachment and entry into host cells [4,5]. It is also a major antigenic determinant that induces generation of neutralizing antibodies and protective immunity at least in human host [8]. Unlike some coronaviruses, in which S protein can be cleaved into two functional subunits, S1 and S2, the S protein of SARS-CoV is not cleavable due to the absence of the proteolytic cleavage site. However, two domains, S1 (residues 14–680) and S2 (residues 681–1,255) were identified in SARS-CoV S protein in the light of their homology with the S1 and S2 subunits [9]. Domain S1 is responsible for binding to angiotensin-converting enzyme-2 (ACE2), which serves as the functional receptor of SARS-CoV [10,11]. Domain S2 mediates viral entry into host cells [12,13]. Previous works indicated that interspecies transmission may be due to the acquisition of mutations in S protein which allows human infection, suggesting that S protein ought to be a major target of selective pressure [6,7,14].

A criterion for the determination of selective pressure is to compare nonsynonymous (amino acid-changing; *d*_*N*_) with synonymous (silent; *d*_*S*_) substitution rates in protein-coding genes. The nonsynonymous/synonymous rate ratio (ω = *d*_*N*_/*d*_*S*_) provides a straightforward measurement of selective pressure at the protein level. The ω values of > 1, 1 and < 1 indicate positive (diversifying) selection, random drift and negative (purifying) selection, respectively. The pairwise analysis showed that S protein, which has an average ω ratio for all amino acid sites greater than 1, is under overall positive selective pressure [7,14,15]. However, the process of adaptation of S protein to human receptors and the positively selected sites associated with this process remain unclear. Because previous epidemic phases [7,14] are unable to adequately reflect the process of animal-to-human transmission and of viral adaptation to human host, we therefore reclassified the SARS groups in the present study. We found that the S protein of SARS-CoV experiences variable positive selective pressures and the positively selected sites are changeable in different epidemic groups. These observations provide a good evidence for understanding the molecular adaptation of SARS-CoV from animals to humans.

## Results

### Positive selection on S genes of SARS-CoV during the whole outbreak from 2002 to 2004

The likelihood values and parameter estimates of 45 S gene sequences from six models implemented in program Codeml are listed in Table 1. The average ω values ranged from 0.36 to 0.69 among all models, showing the evidence of purifying selection. Although the one-ratio model (M0) showed that all sites of S gene have a ω ratio of 0.64, it was easily rejected as a result of the lowest likelihood value (-5818.14) and the likelihood ratio test (LRT) statistic (2 delta lambda statistic, 2Δ*l*) (Table 1). Three models (M2a, M3 and M8) that allow for selection indicated the presence of 5.2–5.9% positively selected sites with similar ω values (9.68–10.29). LRT statistic showed that the three selection models fitted the data significantly better than the null models without selection, supporting the presence of 5.2–5.9% amino acid sites of S gene under strong positive selection (Table 1). At the level of posterior probability > 0.95, four, twelve and eight sites of S protein were identified to be under positive selection (ω > 1) by selection models M2a, M3 and M8, respectively (Table 1). Twelve positively selected sites detected by M3 were also identified by M2a and M8 at the level of posterior probability > 0.9 (Table 1). The number of positively selected sites discovered in the present study was similar to the number of the sites identified in previous reports [16, 17].

### Detection of recombination and positive selection on S genes of SARS-CoV in different epidemic groups

**Figure 1:**
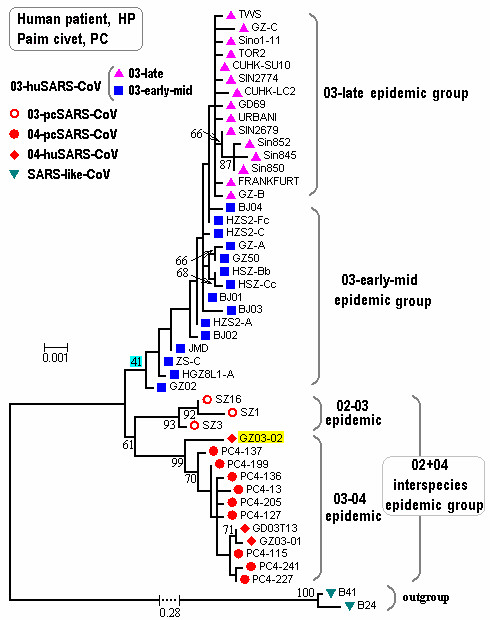
**Phylogenetic tree of 47 S gene sequences from human patients and animals**. The evolutionary process of S proteins during whole epidemic was simplified into three epidemic groups: 02–04 interspecies, 03-early-mid, and 03-late epidemic groups. Each group includes 15 unique S gene sequences after deleting all duplicate sequences (Table S1, see additional file 2). Two sequences isolated from bats were used as the outgroup in phylogenetic tree construction. The tree was constructed by the maximum likelihood method with 1000 bootstrap replicates using PHYML v2.4.4. Only the branch bootstrap values ≥ 50% are shown. PC, palm civet; HP, human patient.

Recombination can influence the detection of positive selection [18,19], and previous studies had proposed that recombination occurs in the origin of SARS-CoV [20]. In 02–04 interspecies epidemic group, human sequence GZ03-02 split 03-pcSARS-CoVs from 04-pcSARS-CoVs, whereas other 04-huSARS-CoVs clustered with 04-pcSARS-CoVs (Fig. 1), suggesting that GZ03-02 may be a recombinant between 03-pcSARS-CoVs and 04-pcSARS-CoVs. However, the bootscan analysis of GZ03-02 using SimPlot software showed that majority of GZ03-02 S gene had the percent of permuted trees less than 40 (Fig. S1, see additional file 1), indicating that they possess similar identity to other sequences, and suggesting that no recombination occurred in this strain [21]. In addition, viral recombination requires the co-infection of different virus strains [22], and there was little chance for GZ03-02 patients to be co-infected with 03-pcSARS-CoV and 04-pcSARS-CoV during 2004 epidemic [7], further supporting the view that no recombination occurred in S gene of GZ03-02 [21].

Three selection models (M2a, M3 and M8) showed that positive selection occurred in both 02–04 interspecies and 03-early-mid epidemic groups (Table 2). For instance, M8 showed that 0.6% of the sites in 02–04 interspecies epidemic group were under positive selection with ω values between 66.0–67.2, and 2.7% of the sites in 03-early-mid epidemic group were under positive selection with ω = 40.9. LRT statistic revealed that three selection models fitted the data better than three null models in both groups of 02–04 interspecies epidemic and 03-early-mid epidemic, which supports further the presence of amino acid sites under positive selection in S protein (Table 2). In contrast, we were unable to identify any site under positive selection with any of the six models in the 03-late epidemic group. Instead, the results for this group were consistent with purifying selection (with ω values of 0.25–0.26) (Table 2).

### Comparison of positively selected sites on S genes in different epidemic groups

The positively selected sites in both groups of 02–04 interspecies epidemic and 03-early-mid epidemic were identified using Codeml program. Although three selection models: M2a, M3 and M8 detected same positively selected sites on S genes, only the results from M8 are shown in Table 3. Four positively selected sites (479, 609, 743, and 765) in 02–04 interspecies epidemic group and four sites (75, 239, 778 and 1163) in 03-early-mid epidemic group were identified at the level of posterior probability > 0.95, respectively. In addition, 25 and 16 sites were detected under positive selection (ω > 1) in 02–04 interspecies epidemic and 03-early-mid epidemic groups at the level of posterior probability > 0.50, respectively. By REL method, completely identical 25 sites in 02–04 interspecies epidemic group and 16 sites in 03-early-mid epidemic group were identified under positive selection at significant level of Bayes factor > 50 (Table 3). When FEL and SLAC methods were used, these sites were also identified under positive selection in despite of not reaching the significant level of p < 0.1. No positively selected site was identified in 03-late epidemic group by three selection models (even at the level of posterior probability > 0.50) implemented in Codeml program and three methods implemented in DataMonkey package (Table 3), indicating that this group was experiencing purifying selection.

In order to investigate the association of positively selected sites with the function of S protein, we compared their location between groups of 02–04 interspecies epidemic and 03-early-mid epidemic. The results show that apart from the site 239, the two groups had completely different sites (Table 3), suggesting for the first time that positively selected sites are variable in different epidemic groups. It was found that 72% (18 out of 25) positively selected sites in 02–04 interspecies epidemic group were located in S1 domain, which is greater than 50% (8 out of 16) of that located in S1 domain in 03-early-mid epidemic group (p = 0.0768) (Table 3). Moreover, 24% of positively selected sites in 02–04 interspecies epidemic group were concentrated in the region of receptor-binding domain (RBD), only 4% in heptad repeat (HR)1-HR2 region (p = 0.0208), but 0% in HR2 region (p = 0.0045). Contrarily, 25% of positively selected sites in 03-early-mid epidemic group were concentrated in HR1–HR2 region (p = 0.0721), 18.8% in HR2 region (p = 0.1425), but only 6.3% in RBD region (Table 3 and 4). These results suggest that positive selection tends to selectively influence certain functions of S protein, but not others in each epidemic group.

### Lineage fixation of positively selected sites on S genes for the adaptation of SARS-CoV to human host

**Figure 2:**
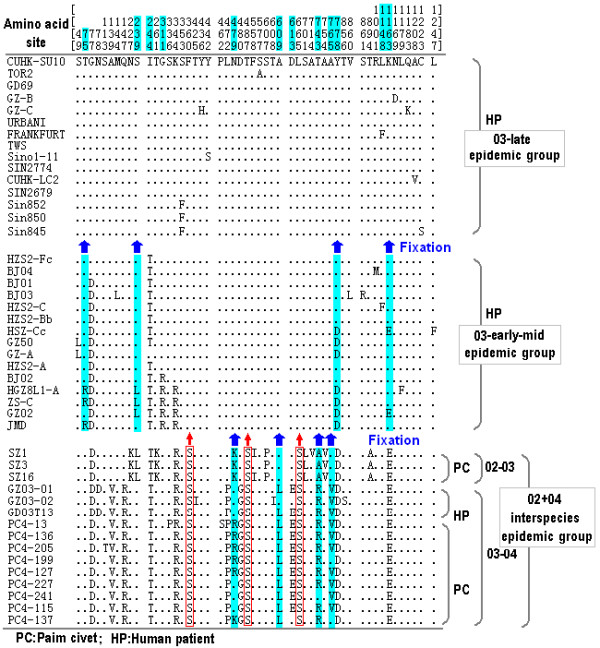
**Amino acid variations of 45 S proteins**. The amino acid sequence of CUHK-SU10 strain isolated from 03-late epidemic group was used as the reference sequence. The shadows represent the sites or residues under positive selection with posterior probability ≥ 0.9. The red sashes highlight three special sites where amino acids were replaced and fixed in all 03 human strains, suggesting selective sweep acting on the S gene.

Four positively selected sites (479, 609, 743 and 765) identified in 02–04 interspecies epidemic group were fixed in 03-early-mid epidemic group (Fig. 2). The 04-pcSARS-CoVs diverged from 03-pcSARS-CoVs after the split between 03-pcSARS-CoVs and 03-huSARS-CoV (Fig. 1) [7]. The comparison of amino acid sequences between 03-pcSARS-CoV and 03-huSARS-CoV suggested that variants N479K and T743A play a dominant role in transition of viral host tropism from animals to humans (Fig. 2). The comparison between 03-huSARS-CoV and 03-pcSARS-CoV sequences discovered two additional variants, L609A and V765A, which may favor viral adaptation to palm civet. Four sites (75T, 239S, 778Y, and 1163K) identified under positive selection in the 03-early-mid epidemic group were fixed in the 03-late epidemic group (Fig. 2). The fixation of these amino acids suggests that they are likely to contribute to the adaptation process of S protein to human receptors.

## Discussion

The S protein of SARS-CoV is responsible for the receptor binding and membrane fusion [10]. It is also a major antigen to stimulate humoral immunity of its host [8]. The amino acid variation of S protein affects virus entry, tissue tropism and host range of SARS-CoV [11,23]. Here, we confirmed that the S gene undergoes strong positive selection [7,14-16], and identified twelve positively selected amino acid sites, including 75, 239, 244, 311, 479, 609, 613, 743, 765, 778, 1148, and 1163 during the whole SARS outbreak (Table 1). Among these sites, positions 239, 311, 479, 609, 743, 778, 1148, and 1163 appeared to be exposed on the surface of S protein [9,24], suggesting that they are likely to play a key role in viral transmission and survival. In addition, it was worth pointing out that SARS-CoV is a rapidly evolving RNA virus with a mutation rate of 0.8–2.38 × 10^-3 ^nucleotide substitution per site per year [25]. The S gene sequences used in the present study were sampled during a year period, and some mutations might be accumulated in late-sampled sequences [26,27]. However, whether the accumulation of these mutations influences the detection of positive selection and the identification of positively selected sites remains unclear [26]. This requires further investigation to confirm.

Adaptation of an animal virus to a new human host usually faces two crucial bottlenecks: the receptor adaptation of viral surface protein to its new host, followed by the adaptation of key enzymes (e.g. viral replicases) associated with viral replication to new cellular components that possibly support poorly productive infection (e.g. non-permissive cells) [21,28]. The latter is not always the step that limits host expansion and most viruses can establish productive infection after their entry of host cells [29]. We found that two key replicases of SARS-CoV, RNA-dependent RNA polymerase (RdRp) and helicase, were not under positive selection (Zhang CY et al., unpublished data), which suggests that receptor adaptation of S protein to human host determines the animal-to-human transmission of SARS-CoV [11,29]. The receptor adaptation of an animal virus to a new human host usually requires two key steps: initial breakthrough of receptor barrier (animal-to-human transmission), followed by the molecular adaptation to human cellular receptors (human-to-human transmission). The two steps together result in eventual establishment of stable infection necessary for efficient spread within human hosts.

In order to better reflect the course of viral trans-species transmission and subsequent adaptation to human hosts, the collection of SARS isolates was reclassified into three epidemic groups: 02–04 interspecies, 03-early-mid, and 03-late epidemic groups in the present study. The 02–04 interspecies epidemic group reflects the process of viral trans-species transmission, and 03-early-mid epidemic group represents the crucial phase of SARS-CoVs to adapt to human host. The two groups correspond with the two key steps described above for a virus to be adapted by a new cellular receptor.

We found that S genes underwent strong positive selection in both groups of 02–04 interspecies epidemic and 03-early-mid epidemic, whereas no positive selection was observed in 03-late epidemic group (Table 2). It suggests that S protein experiences a step-by-step adaptation process to human cellular receptors. On the other hand, the amino acid sites under positive selection in 02–04 interspecies epidemic group differed clearly from those in 03-early-mid epidemic group, suggesting for the first time the changes in positively selected sites in different epidemic groups. It was reported previously that two functional domains S1 and S2 of SARS-CoV S protein are responsible for receptor recognition and membrane fusion, respectively [10]. In domain S1, RBD has been demonstrated to concentrate in a 193-amino acid fragment (residues 318–510), which is adequate for binding to human ACE2 [30]. In domain S2, two highly conserved regions HP1 (residues: 889–972) and 2 (residues: 1142–1185) are crucial for membrane fusion [31]. Importantly, a larger proportion of positively selected sites was located in RBD than in HR1–HR2 or HR2 regions in 02–04 interspecies epidemic group, and a greater percentage of these sites occurred in HR1–HR2 region than in RBD in 03-early-mid epidemic group (Fisher's exact test: p = 0.045 for HR1–HR2 vs. RBD, and p = 0.033 for HR2 vs. RBD) (Table 4). These differences suggest that positive selection prefers influencing the receptor-binding function in 02–04 interspecies epidemic group, and then associates with the membrane fusion function in 03-early-mid epidemic group.

In 02–04 interspecies epidemic group, four positively selected sites 479, 609, 743 and 765 appeared to be fixed in 03-early-mid epidemic group. Comparing the amino acid sequences of S genes from 03-huSARS-CoV and 03-pcSARS-CoV, we discovered that two positively selected amino acid substitutions (N479K and T743A) are likely to play a role in viral receptor switch. Previous *in vitro *experiments proved that the residues at positions 479 and 487 are important determinants for SARS-CoV cell tropism and animal-to-human transmission [11,23,32,33]. Residue 479N (asparagine) increases the affinity of S protein to human receptor by specifically interacting with the residue 34H (histidine) of ACE2, which is present in human but not in palm civet ACE2 [11,32]. Recently, Zheng et al. reported that active peptide (residues 737–756) of S protein which contains residue 743T (threonine) effectively inhibited the entry of huSARS-CoV into human cells [34]. The corresponding peptide with residue 743A (alanine) from 03-pcSARS-CoV appeared less potent against huSARS-CoV [34]. These observations suggested that residue 743 may influence viral receptor tropism via a way different from ACE2 binding [34]. Besides ACE2, human DC-SIGN and DC-SIGNL were also shown to be able to enhance SARS-CoV infection by a non-receptor mechanism [35,36]. However, the influence of residue 743 on potential interaction between S protein and DC-SIGN or DC-SIGNL remains unknown. On the other hand, of the four positively selected sites, two variants L609A and V765A, were observed in most 04-pcSARS-CoVs, but not in 03-pcSARS-CoVs, suggesting that they may contribute to viral adaptive evolution from 03-pcSARS-CoV to 04-pcSARS-CoV.

The sequence comparison between groups of 02–04 interspecies epidemic and 03-early-mid epidemic allowed us to find additional three special substitutions (T1079C, C1460G and T1994C), which lead to three replacement (F360S, T487S and L665S) (Fig. 2). Of particular importance was that all three sites were monomorphic for serine in the 02–04 interspecies epidemic group, and utterly monomorphic for phenylalanine at site 360, threonine at 487 and leucine at 665 in the 03-human epidemic group (including 03-early-mid and 03-late phases) (Fig. 2). Previous studies revealed that the replacement T487S benefits the receptor switch of SARS-CoVs from palm civets to human [11,23,32]. However, both Codeml program and DataMonkey package did not detect positive selection on this site. Therefore, the best explanation should be that the selective sweep also drives the adaptive evolution of S gene from animals to humans, despite of little experimental evidence supporting the advantageous mutations in sites 360 and 665.

After breaking through the interspecies receptor barrier, SARS-CoV spread quickly among human hosts and formed the 03-early-mid epidemic group, a very important period for viral adaptation to human host. During this stage, four major sites (75, 239, 778 and 1163) of S protein were identified under positive selection. Among them, sites 778 and 1163 were located in the S2 domain of S protein, suggesting that they should associate with membrane fusion, less probability with receptor recognition [11,23]. The S2 domain contains two highly conserved heptad repeats HP1 and HP2, both of them form a six-helix bundle structure via hydrophobic interaction, facilitating membrane fusion [12,31]. At site 1163 of HR2 region, the positively charged residue lysine replaced the negatively charged residue glutamic acid in 03-early-mid epidemic group, suggesting that residue lysine may be advantageous for S protein adaptation to human cellular receptors. The antiviral research showed that active peptide (residues 1161–1180) with residue1163E derived from animal virus possesses less inhibitory activity against human SARS-CoVs than its human virus counterpart of 1163K [34], which supports the view that the substitution K1163E contributes to adaptation of SARS-CoV to human cellular receptors. On the other hand, a strong neutralizing epitope containing residues 1055–1192 also implicated the possible role of residue 1163 in induction of neutralizing antibodies despite of little evidence available for its action on antibody escape [37,38]. As for residue 778, uncharged amino acid tyrosine was fixed in the 03-late epidemic group by replacing the negatively charged residue aspartic acid. The importance of residue 778 cannot be assessed until further site-directed mutagenesis research is conducted. With respect to residues 75 and 239, they are unlikely to participate in the viral adaptation process due to their location outside RBD and S2 domain regions.

## Conclusion

A total of 12 sites (75, 239, 244, 311, 479, 609, 613, 743, 765, 778, 1148 and 1163) in S protein were detected under positive selective pressure. Among them, 8 sites are exposed on the surface of S protein. It was also found that the S protein of SARS-CoV experiences variable positive selective pressures before reaching stabilization, and the positively selected sites are changeable in different epidemic groups. More importantly, a larger proportion of positively selected sites identified in 02–04 interspecies epidemic group was located in RBD region of S protein, suggesting that receptor binding function is predominant at this stage. On the other hand, more positively selected sites were located in HR1–HR2 region in 03-early-mid epidemic group, suggesting that the membrane fusion function becomes a major task in association with positive selection at this period. The variation of positive selective pressures and positively selected sites of S protein provide a valuable evidence for understanding the molecular adaptation of S protein from animals to humans.

## Methods

### Sequences

A total of 102 *Spike *(S) gene sequences of SARS-CoVs and SARS-like-CoVs from human and animals were retrieved from the GenBank (Table S1, see additional file 2). Among them, 100 S gene sequences were obtained from five previously classified epidemic phases: 02–03, 03-early, 03-middle, 03-late and 03–04 epidemic phases, which represent the SARS outbreak sequence from later 2002 to early 2004 [7,14]. The 02–03 epidemic phase contained SARS-like-CoV sequences from animals (03-pcSARS-CoV) [6]. The strains of 03-early, 03-middle and 03-late phases were isolated from human (03-huSARS-CoV). The 03–04 epidemic phase represented the re-emergence of SARS in human patients (04-huSARS-CoV) and palm civets (04-pcSARS-CoV) [7]. The other two S sequences of SARS-like-CoVs isolated from bats were used as the outgroup of phylogenetic tree [39,40].

### Phylogenetic analysis, tree construction and recombination analysis

After deletion of identical sequences, only 45 distinctive sequences out of 100 S sequences were used for phylogenetic analysis (Table S1, see additional file 2). They were aligned together with two outgroup sequences using CLUSTAL × (Ver. 1.83) [41]. The phylogenetic tree of S gene was obtained by using ML (maximum likelihood) (PHYML v2.4.4) [42] and NJ (neighbor-joining) (MEGA 3.0) [43] methods, and the reliability of the trees was evaluated by the bootstrap method with 1,000 replications. The *d*_*N*_/*d*_*S *_value was used to detect positive selection. Since recombination of genes can result in artificially high *d*_*N*_/*d*_*S *_values and a false detection of positive selection [18,19], the SimPlot Version 3.5.1 [44] was applied to determine whether the recombination occurs in S gene of SARS-CoV.

### Re-classification of SARS epidemic phases

The outbreak of SARS was previously divided into five epidemic phases: 02–03 epidemic, 03-early, 03-middle, 03-late, and 03–04 epidemic phases based on the epidemiological investigation [7,14]. The tree topology showed that isolates from the 02–03 epidemic, 03-human epidemic and 03–04 epidemic phases formed a monophyletic clade, respectively (Fig. 1) [7]. The clades formed by 03-pcSARS-CoVs and 04-pcSARS-CoVs clustered together, diverging from the clade formed by 03-huSARS-CoVs, which have been demonstrated being responsible for transmission of SARS-CoVs from animals such as palm civets to human [6,7,14]. These suggested that the origin of 03-huSARS-CoVs should be a virus strain, which was prevalence in palm civets or other animals before November 2002, but different from both 03-pcSARS-CoV and 04-pcSARS-CoV [6,7,25].

Because the 02–03 epidemic phase included SARS-CoV sequences from animals (palm civets and a raccoon dog) and the 03–04 epidemic phase contained 04-huSARS-CoV and 04-pcSARS-CoV, they should be at least partially reflect the course of viral interspecies transmission. In order to more realistically detect the adaptive evolution of S genes in interspecies transmission, we merged 02–03 and 03–04 epidemic phases into a unique epidemic group: 02–04 interspecies epidemic group, representing the course of SARS-CoV interspecies transmission. With respect to 03-human epidemic, it was divided into three phases in the previous studies. In the current study, it formed a monophyletic clade in phylogenetic tree with a low bootstrap value (41%) support (Fig. 1). In fact, the sequences from 03-early and 03-middle phases clustered together, and did not split each other. In addition, pairwise comparison of S gene sequences demonstrated that 03-early and 03-middle phases under positive selection, suggesting that both phases associate with the adaptation to human receptor [14]. On the other hand, the 03-late epidemic phase experienced a longer epidemic time (more than four months) than the sum of 03-early and 03-middle epidemic phases (about three months), and showed a lower sequence divergence [14]. We therefore divided 03-human epidemic into two groups: 03-early-mid and 03-late epidemic groups according to the epidemiological investigation and their epidemic time. Thus, the evolutionary process of S proteins during the whole SARS epidemic was simplified into three epidemic groups: 02–04 interspecies, 03-early-mid and 03-late epidemic groups in the present study (Fig. 1). Each group might represent a unique epidemic phase in the whole SARS outbreak including initial animal-to-human transmission phase, adaptation to human receptor phase and subsequent lineage fixation phase, respectively.

### Analysis of the adaptive evolution and identification of positively selected sites

The program Codeml implemented in the PAML 3.14 b software package was used to investigate the adaptive evolution of S protein [45]. A total of 45 aligned S gene sequences, isolated from the different epidemic groups, were selected to test whether they were under positive selection in the whole outbreak. Six models of codon substitution, M0 (one-ratio), M1a (NearlyNeutral), M2a (PositiveSelection), M3 (discrete), M7 (beta), and M8 (beta and ω) were used in the analysis [46]. M0 assumes that all sites have the same ω ratio. M1a assumes two classes of sites in proteins in proportions p0 and p1 (1 – p0) with 0 < ω0 < 1 (purifying selection) and ω1 = 1 (neutral sites). M2a adds a proportion (p2) to account for a class of sites where w2 is estimated from the data and can be > 1. M3 uses a general discrete distribution with three site classes, with the proportions (p0, p1 and p2) and the ω ratios (ω0, ω1 and ω2) estimated from the data. M7 assumes a beta distribution (p, q) for 10 different ω ratios in the interval (0, 1). M8 adds an extra class of sites with positive selection (ω > 1) to the beta (M7) model [46,47]. Therefore, the null models M0, M1a and M7 fix the ω ratios between 0 and 1, and do not allow the presence of positively selected sites. The alternative models M2a, M3 and M8 account for positive selection by using parameters, which estimate ω greater than 1, and allow for the variable ω along codon sequence.

Likelihood ratio test (LRT) [48] was performed for detecting the presence of positively selected sites by comparing the models which do not allow for positive selection with the models which allow for positive selection. The LRT was performed by taking twice the difference in log likelihood between nested models, and testing for significance using the χ^2 ^distribution with the degrees of freedom (d.f.) equivalent to the difference in the number of parameters between models. If the LRT is significant, positive selection is inferred. In the present study, three LRTs (M0 vs. M3, M1a vs. M2a, and M7 vs. M8) were used to detect positive selection. The Bayes empirical Bayes (BEB) approach implemented in M2a and M8 was used to determine the positively selected sites by calculating the posterior probabilities (p) of ω classes for each site [47]. The sites with high posterior probabilities (p > 0.95) coming from the class with ω > 1 were believed to be under positive selection [46].

Because positive selection on S gene was detected during the whole SARS outbreak, the sequences from three different epidemic groups were also analyzed using Codeml program to further specify the epidemic group in which positive selection occurred and the exact location of positively selected sites in S proteins. To further confirm positively selected sites identified by using program Codeml, three additional methods including single likelihood ancestor counting (SLAC), fixed effects likelihood (FEL), and random effects (REL) as implemented in the on-line DataMonkey package were employed [49,50]. To detect positively selected sites, the 0.1 level of significance was used for both SLAC and FEL, and default significant level of Bayes factor > 50 was used for REL. In the process of analysis, the results of three epidemic groups were obtained. However, as a result of the large amount of data, which exceed the acceptability of DataMonkey package, we failed to obtain the results of 45 sequences, which represent the whole SARS outbreak. In order to reduce the influence of type-I error rate (false discovery rate) [51], the sites identified under positive selection at significant level by both Codeml program and REL methods in DataMonkey package were used to investigate their lineage fixation for the adaptation of SARS-CoV S protein from animals to human hosts.

### Statistical analysis

The statistical analysis for the comparison of positively selected sites between different groups was performed using one-sided χ^2 ^test or one-sided Fisher's exact test in GraphPad Prism version 4.03 for Windows demo.

## Authors' contributions

CYZ and JFW conceived and designed the study, performed the collection and evolutionary analysis of the data. CYZ drafted the manuscript and JFW and SHH revised the manuscript. SHH supervised and coordinated the whole project. All authors have read and approved the final manuscript.

## Supplementary Material

Additional file 1 **Figure S1. Bootscanning analyses of S gene sequences of GZ03-02 for detecting recombination**. The bootstrap values are plotted for a window of 200 bp moving in increments of 20 bp along the alignment. Click here for file

Additional file 2 **Table S1. List of GenBank accession numbers for 102 S gene sequences of SARS-CoVs analyzed in the text**. The 02–03 epidemic phase includes three isolates (SZ1, SZ3 and SZ16) from palm civets and one (SZ13) from a dog. During 03–04 epidemic phase, the strains were mainly isolated from palm civets, only three (GD03T13, GZ03-01 and GZ03-02) were isolated from human patients. All strains of 03-early-mid epidemic and 03-late epidemic groups were isolated from human patients. Two outgroup sequences (B24 and B41) were isolated from Chinese horseshoe bats. Click here for file

## Figures and Tables

**Table 1:** Likelihood values and parameter estimates for 45 S gene sequences from the whole epidemic of SARS from 2002 to 2004.

**Model code**	**lnL**	***d*_N_/*d*_S_**	**Estimates of parameters**	**2Δ*l***	**Positively selected sites**
M0 (one-ratio)	-5818.14	0.64	ω = 0.64	51.91	None
M3 (discrete)	-5792.19	0.69	*p*_0 _= 0.43340, *p*_1 _= 0.50712 (*p*_2 _= 0.05948), ω_0 _= 0.12, ω_1 _= 0.13, ω_2 _= 9.68	(13.28)	**75T 239S 244T 311G 479R 609L 613E 743R 765V 778D 1148L 1163E**
M1a (NearlyNeutral)	-5807.78	0.36	*p*_0 _= 0.63944 (*p*_1 _= 0.36056)	31.25	Not allowed
M2a (PositiveSelection)	-5792.16	0.69	*p*_0 _= 0.78594, *p*_1 _= 0.16251 (*p*_2 _= 0.05155), ω_2 _= 10.29	(9.21)	**75T 239S **244T 311G **479R **609L 613E **743R **765V 778D 1148L 1163E
M7 (beta)	-5807.92	0.40	*p *= 0.00568, *q *= 0.00856	31.51	Not allowed
M8 (beta&v)	-5792.16	0.69	*p*_0 _= 0.94614 (*p*_1 _= 0.05386) *p *= 0.05787, *q *= 0.30879, ω = 10.11	(9.21)	**75T 239S **244T **311G 479R 609L **613E **743R **765V 778D **1148L 1163E**

The values in parentheses represent the significant level of 0.01 with a χ^2^distribution at d.f. = 4 (M0 vs. M3) or 2 (M1a vs. M2a and M7 vs. M8). The amino acid sequence of strain PC4-13 was used as the sequence reference, and positive selected sites were identified with posterior probability p ≥ 0.9. In boldface, p ≥ 0.95.

**Table 2:** Phylogenetic analysis by ML estimation for SARS S gene sequences from different epidemic groups.

**Epidemic phases**	**Model code**	**lnL**	***d*_N_/*d*_S_**	**Estimates of parameters**	**2Δ*l***	**Positive selection**
**02–04 interspecies epidemic group**	M0 (one-ratio)	-5339.90	0.64	ω = 0.64	40.23	Yes
	M3 (discrete)	-5319.78	0.77	*p*_0 _= 0.00000, = p_1 _= 0.99407 (*p*_2 _= 0.00593), ω_0 _= 0.00, ω_1 _= 0.38, ω_2 _= 66.02	(13.28)	
	M1a (NearlyNeutral)	-5334.74	0.35	*p*_0 _= 0.65391 (*p*_1 _= 0.34609)	29.92	Yes
	M2a (PositiveSelection)	-5319.78	0.77	*p*_0 _= 0.99407, *p*_1 _= 0.00000 (*p*_2 _= 0.00593), ω_2 _= 66.01	(9.21)	
	M7 (beta)	-5336.08	0.30	*p *= 0.01702, *q *= 0.03977	32.23	Yes
	M8 (beta&v)	-5319.97	0.80	*p*_0 _= 0.99405 (*p*_1 _= 0.00595), *p*_0 _= 0.01217, *q *= 0.01714 ω = 67.24	(9.21)	
						
**03-early-mid epidemic group**	M0 (one-ratio)	-5194.47	0.94	ω = 0.94	24.25	Yes
	M3 (discrete)	-5182.34	1.10	*p*_0 _= 0.05053, *p*_1 _= 0.92251 (*p*_2 _= 0.02696), ω_0 _= ω_1 _= 0.00, ω_2 _= 40.88	(13.28)	
	M1a (NearlyNeutral)	-5192.6 9	0.45	*p*_0 _= 0.54981 (*p*_1 _= 0.45019)	20.70	Yes
	M2a (PositiveSelection)	-5182.34	1.10	*p*_0 _= 0.97303, *p*_1 _= 0.00000 (*p*_2 _= 0.02696), ω_2 _= 40.88	(9.21)	
	M7 (beta)	-5192.73	0.40	*p *= 0.00510, *q *= 0.00776	20.78	Yes
	M8 (beta&v)	-5182.34	1.10	*p*_0 _= 0.97303 (*p*_1 _= 0.02697), *p *= 0.00500, *q *= 1.39962, ω = 40.87	(9.21)	
						
**03-late epidemic group**	M0 (one-ratio)	-5121.66	0.26	ω = 0.26	NA	No
	M3 (discrete)	-5121.66	0.26	*p*_0 _= 0.43056, *p*_1 _= 0.37490 (*p*_2 _= 0.19455), ω_0 _= 0.25, ω_1 _= 0.26, ω_2 _= 0.26	(13.28)	
	M1a (NearlyNeutral)	-5121.66	0.26	*p*_0 _= 1.00000 (*p*_1 _= 0.00000)	NA	No
	M2a (PositiveSelection)	-5121.66	0.26	*p*_0 _= 1.00000, *p*_1 _= 0. 00000 (*p*_2 _= 0.00000), ω_0 _= 0.26, ω_1 _= ω_2 _= 1.00	(9.21)	
	M7 (beta)	-5121.66	0.25	*p *= 33.88884, *q *= 99.00000	NA	No
	M8 (beta&v)	-5121.68	0.26	*p*_0 _= 1.00000 (*p*_1 _= 0.00000), *p *= 0.64576, *q *= 1.83663, ω = 2.08	(9.21)	

The values in parentheses represent the significant level of 0.01 with a χ^2^distribution at d.f. = 4 (M0 vs. M3) or 2 (M1a vs. M2a and M7 vs. M8). NA, not applicable.

**Table 3:** Positively selected sites identified by Codeml program and REL in DataMonkey package.

Domains	**Positively selected sites in different epidemic groups**		
	
	**02–04 interspecies**	**03-early-mid**	**03-late**
**S1**	78 113 139 147 227 239 261 336 425 462 472 **479 **480 558 607 608 **609 **613	49 **75 **77 144 **239 **244 311 344	None
**S2**	701 714 **743 **754 **765 **856 894	**778 **860 861 1001 1148 **1163 **1179 1247	None

Positively selected sites identified by program Codeml at the level of posterior probability ≥ 0.95 are shown in boldface. The underlines represent the sites locating in receptor-binding domain (RBD) (residues: 318–510) of S1 domain or in heptad repeat (HR)1 (residues: 889–972)-HR2 (residues: 1142–1185) region of S2 domain.

**Table 4:** The number of positively selected sites in different functional domains of S protein.

**Functional domain**	**02–04 interspecies epidemic group**	**03-early-mid epidemic group**	**Fisher's exact test (p)**
**RBD**	6	1	
**HR1–HR2**	1	4	0.045455
**HR2**	0	3	0.033333

The Fisher's exact test was performed for HR1–HR2 vs. RBD, and HR2 vs. RBD, respectively.
