# Supplementary material for: Adaptive evolution of the spike gene of SARS coronavirus: changes in positively selected sites in different epidemic groups
Source: BMC Microbiol. 2006 Oct 4;6:88. doi: 10.1186/1471-2180-6-88 (PMC1609170; doi:10.1186/1471-2180-6-88)
Supplement: Additional file 1 — Figure S1. Bootscanning analyses of S gene sequences of GZ03-02 for detecting recombination. The bootstrap values are plotted for a window of 200 bp moving in increments of 20 bp along the alignment. [file 1471-2180-6-88-S1.doc]

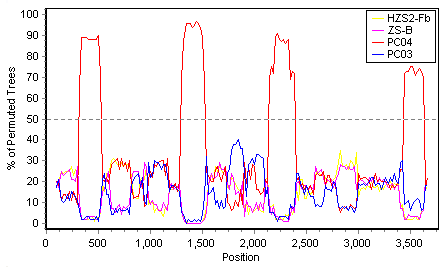


**Additional file 1 –Figure S1. Bootscanning analyses of S gene sequences of GZ03-02 for detecting recombination**. The bootstrap values are plotted for a window of 200 bp moving in increments of 20 bp along the alignment.
