# Supplementary material for: Adaptive evolution of the spike gene of SARS coronavirus: changes in positively selected sites in different epidemic groups
Source: BMC Microbiol. 2006 Oct 4;6:88. doi: 10.1186/1471-2180-6-88 (PMC1609170; doi:10.1186/1471-2180-6-88)
Supplement: Additional file 2 — Table S1. List of GenBank accession numbers for 102 S gene sequences of SARS-CoVs analyzed in the text. The 02–03 epidemic phase includes three isolates (SZ1, SZ3 and SZ16) from palm civets and one (SZ13) from a dog. During 03–04 epidemic phase, the strains were mainly isolated from palm civets, only three (GD03T13, GZ03-01 and GZ03-02) were isolated from human patients. All strains of 03-early-mid epidemic and 03-late epidemic groups were isolated from human patients. Two outgroup sequences (B24 and B41) were isolated from Chinese horseshoe bats. [file 1471-2180-6-88-S2.doc]

**Additional file 2 –Table S1. List of GenBank accession numbers for 102 S gene sequences of SARS-CoVs analyzed in the text.**

| **Epidemic phases** | | Strains | **Accession number** | **Epidemic phases** | Strains | **Accession number** |
| --- | --- | --- | --- | --- | --- | --- |
| **02-04 interspecies epidemic group**  (17 sequences) | **02-03 phase** | SZ1 | AY304489 | **03-late epidemic group**  (56 sequences) | GZ-B | AY394978 |
|  |  | SZ3 | AY304486 |  | GZ-C | AY394979 |
|  |  | SZ13 | AY304487 |  | TOR2 | AY274119 |
|  |  | SZ16 | AY304488 |  | URBANI | AY278741 |
|  |  |  |  |  | WHU | AY394850 |
|  | **03-04 phase** | PC4-13 | AY613948 |  | HKU-39849 | AY278491 |
|  |  | PC4-115 | AY627044 |  | CUHK-SU10 | AY282752 |
|  |  | PC4-127 | AY613951 |  | CUHK-LC2 | AY394999 |
|  |  | PC4-136 | AY613949 |  | CUHK-LC4 | AY395001 |
|  |  | PC4-137 | AY627045 |  | FRANKFURT | AY291315 |
|  |  | PC4-145 | AY627046 |  | TW3 | AY502926 |
|  |  | PC4-199 | AY627047 |  | TW8 | AY502931 |
|  |  | PC4-205 | AY613952 |  | TW10 | AY502923 |
|  |  | PC4-227 | AY613950 |  | TW11 | AY502924 |
|  |  | PC4-241 | AY627048 |  | TWH | AP006557 |
|  |  | GD03T13 | AY525636 |  | TWK | AP006559 |
|  |  | GZ03-01 | AY568539 |  | TWS | AP006560 |
|  |  | GZ03-02 | AY613947 |  | Sino1-11 | AY485277 |
|  |  |  |  |  | Sino3-11 | AY485278 |
| **03-early-mid epidemic group**  (27 sequences) | | GZ02 | AY390556 |  | Sin845 | AY559093 |
|  |  | HGZ8L1-A | AY394981 |  | Sin849 | AY559086 |
|  |  | ZS-A | AY394997 |  | Sin850 | AY559096 |
|  |  | ZS-B | AY394996 |  | Sin852 | AY559082 |
|  |  | ZS-C | AY395003 |  | SIN2677 | AY283795 |
|  |  | HSZ-Bb | AY394985 |  | SIN2748 | AY283797 |
|  |  | HSZ-Bc | AY394994 |  | Sin3765V | AY559084 |
|  |  | HSZ-Cb | AY394986 |  | TC1 | AY338174 |
|  |  | HSZ-Cc | AY394995 |  | TC2 | AY338175 |
|  |  | HGZ8L1-B | AY394982 |  | TC3 | AY348314 |
|  |  | GZ50 | AY304495 |  | AS | AY427439 |
|  |  | GZ-A | AY394977 |  | A11S | AY345986 |
|  |  | JMD | AY394988 |  | A7N | AY345987 |
|  |  | BJ01 | AY278488 |  | STL2 | AY345988 |
|  |  | BJ02 | AY278487 |  | TW1 | AY291451 |
|  |  | BJ03 | AY278490 |  | TW2 | AY502925 |
|  |  | BJ04 | AY279354 |  | TW4 | AY502927 |
|  |  | CUHK-W1 | AY278554 |  | TW5 | AY502928 |
|  |  | HZS2-A | AY394983 |  | TW6 | AY502929 |
|  |  | HZS2-Bb | AY395004 |  | TW7 | AY502930 |
|  |  | HZS2-C | AY394992 |  | TW9 | AY502932 |
|  |  | HZS2-D | AY394989 |  | TWC | AY321118 |
|  |  | HZS2-E | AY394990 |  | TWJ | AP006558 |
|  |  | HZS2-Fb | AY394987 |  | TWY | AP006561 |
|  |  | HZS2-Fc | AY394991 |  | GD69 | AY313906 |
|  |  | HGZ8L-2 | AY394993 |  | HSR | AY323977 |
|  |  | NS-1 | AY508724 |  | Sin847 | AY559095 |
|  | |  |  |  | Sin848 | AY559085 |
| Outgroup (2 sequences) | | B24 | DQ022305 |  | SIN2774 | AY283798 |
|  |  | B41 | DQ084199 |  | SIN2500 | AY283794 |
|  | |  |  |  | PUMC01 | AY350750 |
|  | |  |  |  | PUMC02 | AY357075 |
|  | |  |  |  | PUMC03 | AY357076 |
|  | |  |  |  | SIN2679 | AY283796 |
|  | |  |  |  | CUHK-LC1 | AY394998 |
|  | |  |  |  | CUHK-LC3 | AY395000 |
|  | |  |  |  | CUHK-LC5 | AY395002 |

**NOTE:** The 02-03 epidemic phase includes three isolates (SZ1, SZ3 and SZ16) from palm civets and one (SZ13) from a dog. During 03-04 epidemic phase, the strains were mainly isolated from palm civets, only three (GD03T13, GZ03-01 and GZ03-02) were isolated from human patients. All strains of 03-early-mid epidemic and 03-late epidemic groups were isolated from human patients. Two outgroup sequences (B24 and B41) were isolated from Chinese horseshoe bats.
